# Current Trends in Clinico-Bacteriological Profile and Antimicrobial Susceptibility Pattern in Active Chronic Suppurative Otitis Media (Safe and Unsafe) at a Tertiary Care Center in Uttarakhand: An Observational Study

**DOI:** 10.7759/cureus.69525

**Published:** 2024-09-16

**Authors:** Monika Sammal, Bhawana Pant, Nidhi Negi, Vikas Sikarwar

**Affiliations:** 1 Department of Otolaryngology and Head and Neck Surgery, Government Doon Medical College, Dehradun, IND; 2 Department of Microbiology, Government Doon Medical College, Dehradun, IND

**Keywords:** antibiotic susceptibility, antimicrobial resistance, bacteriological, chronic otitis media, ear discharge

## Abstract

Introduction: Otitis media is defined as inflammation of the middle ear due to any cause that may also involve any contiguous pneumatized portion of the temporal bone. It is also one of the most common diseases of childhood after viral upper respiratory tract infection. As a result of the widespread availability of over-the-counter topical antibiotics and the irrational use of these agents, there is a development of multidrug-resistant bacteria.

Aim: The aim of this study was to assess the current bacteriological profile and antimicrobial susceptibility pattern in clinically diagnosed cases of active chronic otitis media (COM) at a tertiary care center in Uttarakhand.

Materials and methods: The proposed observational cross-sectional study was conducted in the Department of Otolaryngology and Head and Neck Surgery and Department of Microbiology of Government Doon Medical College Hospital, Dehradun, over a period of 18 months from August 2022 to January 2024. One hundred and thirty-seven cases of active COM fulfilling the inclusion criteria were recruited in the study. Under strict aseptic conditions, pus samples were collected from the middle ear using two sterile cotton swabs under microscopic examination and sent to the microbiology lab for pathogen identification and isolation. The isolated organisms were subjected to antibiotic susceptibility testing using VITEK-2 as per the latest Clinical and Laboratory Standards Institute (CLSI) guidelines.

Results: In the present study, the most common age group affected by COM was between 18 and 30 years with female predominance. *Pseudomonas aeruginosa* (33.6%) was the predominantly isolated organism followed by *Staphylococcus aureus* (17.8%). Considering the antibiotic susceptibility, *Pseudomonas aeruginosa* was susceptible to piperacillin/tazobactam and meropenem while least sensitive to levofloxacin and ciprofloxacin. *Staphylococcus aureus* was susceptible to linezolid, daptomycin, and vancomycin. The maximum resistance of *Staphylococcus aureus* was observed to ciprofloxacin and levofloxacin.

Conclusion: The study of changing clinico-bacteriological profile and antibiotic susceptibility pattern in cases of COM due to variations in the geography and environment of the study population can help to guide appropriate antibiotic treatment and thus prevent the development of multidrug-resistant bacteria.

## Introduction

Otitis media is one of the most common diseases of childhood and a global public health problem leading to hearing impairment particularly in developing countries. Chronic otitis media (COM) refers to the presence of intractable pathology of greater than three months duration within the middle ear system in the setting of a permanent tympanic membrane defect. Retraction pockets, atelectasis, and perforations secondary to trauma, infection, and surgery comprise the defect. The term chronic suppurative otitis media (CSOM) is used in the presence of persistent ear discharge of more than three months duration [1]. Mucosal polyps and thickened granular middle ear mucosa are typical characteristic findings of CSOM [2]. Lack of hygiene, nutritional deficiency, overcrowding, recurrent upper respiratory tract infection, and poor access to healthcare facilities are some of the critical risk factors for the development of COM [3]. As per the World Health Organization (WHO), an estimated 65-330 million people worldwide are affected by CSOM, of whom 50% suffer from hearing impairment [4]. In cases of COM, *Pseudomonas aeruginosa*, *Staphylococcus aureus*, and other Gram-negative bacilli (e.g., *Escherichia coli*, *Proteus* species (spp.), and *Klebsiella* spp.) are the most commonly isolated aerobic bacteria. The external auditory canal has a moist environment, which serves as a residing site for *Pseudomonas aeruginosa*, whereas *Staphylococcus aureus *is present within the human nares. Topical antibiotics with or without steroids [2] are used for the conservative management of infected perforations. Antibiotics should be directed against the most common causative organism, such as *Pseudomonas aeruginosa* and *Staphylococcus aureus*. Culture-directed antibiotics should be used in case of recurrent or chronic infection in which cultures should be obtained from the middle ear to avoid possible contaminating flora, particularly *Pseudomonas aeruginosa* from the external auditory canal [1,5]. The study of changing clinico-bacteriological profiles and antibiotic susceptibility patterns in cases of COM due to variations in the geography and environment of the study population can help to guide appropriate antibiotic treatment. This also helps to prevent the development of multidrug-resistant bacteria [6,7]. Only a few studies have been conducted in the past in the state of Uttarakhand, with the focus primarily on the hilly areas of the state. Hence, this present study was done with the intention of observing the bacteriological profiles and antibiotic susceptibility in patients suffering from CSOM and attending this tertiary care center in the northern part of the country.

## Materials and methods

This observational cross-sectional study was conducted in the Department of Otolaryngology and Head and Neck Surgery and Department of Microbiology of Government Doon Medical College Hospital, Dehradun, over a period of 18 months from August 2022 to January 2024. The study was approved by the Institutional Ethics Committee of Government Doon Medical College Hospital (approval number: GDMC/IEC/2023/15), and informed consent was taken from patients enrolled in this study.

Inclusion and exclusion criteria

All clinically diagnosed cases of active COM between 18 and 60 years of age, irrespective of gender, giving consent for the study were included in the study. In contrast, any acute infections of the external ear, middle ear, and inner ear, operated cases of COM, fungal infection and malignancy of the external and middle ear, and patients on topical and systemic antibiotics for the last one week were excluded from the study. 

The sample size was decided using the formula n=Zα^2^ pq/d^2 ^where n is the sample size, Zα is the Z score at a 5% level of significance, p is the prevalence which is 91.18 [8], q is (100-p), and d is the margin of error (5-20% of p). With 95% confidence level or power and taking 5% as the margin of error, 137 is the calculated sample size.

A history and complete clinical examination of subjects was done, and a provisional diagnosis was made. Examination under a microscope was done in every case to confirm the clinical findings. The pinna was cleaned using a spirit swab followed by suction cleaning of pus from the cartilaginous and outer half of the bony auditory canal. Using microscopic examination and aural speculum, pus samples for culture and sensitivity were collected from the middle ear under strict aseptic conditions using two sterile cotton swabs. The sample was sent to the microbiology lab for Gram stain and subsequently processed for the isolation of pathogens by inoculating into blood agar, chocolate agar, and MacConkey agar. The culture plates were incubated at 37°C, and the presence of growth at the end of 24 hours and 48 hours was observed. At the end of 48 hours, plates showing no growth were reported as culture negative. Plates showing growth were processed further for pathogen identification using appropriate biochemical reactions. Isolates were subjected to antibiotic susceptibility testing by VITEK-2 as per the latest Clinical and Laboratory Standards Institute (CLSI) guidelines [9]. 

Statistical analysis

All the data was tabulated in a Microsoft Excel worksheet, and IBM SPSS Statistics for Windows, Version 22.0 (Released 2013; IBM Corp., Armonk, New York, United States), was used for the analysis of the data. Descriptive statistical methods were used to represent the data as tables, and categorical variables were defined using frequencies and percentages.

## Results

**Figure 1:**
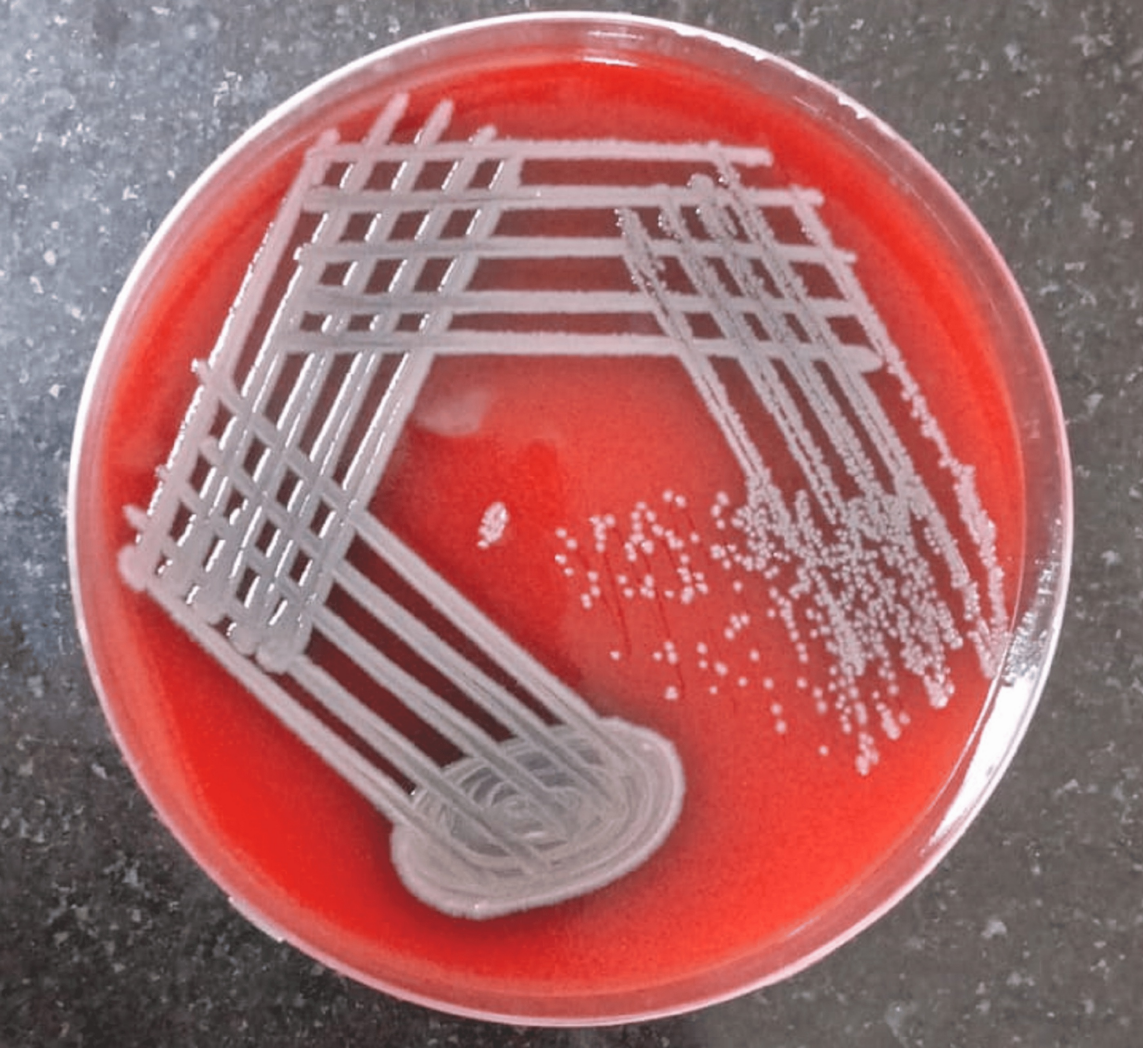
Pseudomonas aeruginosa on blood agar

**Figure 2:**
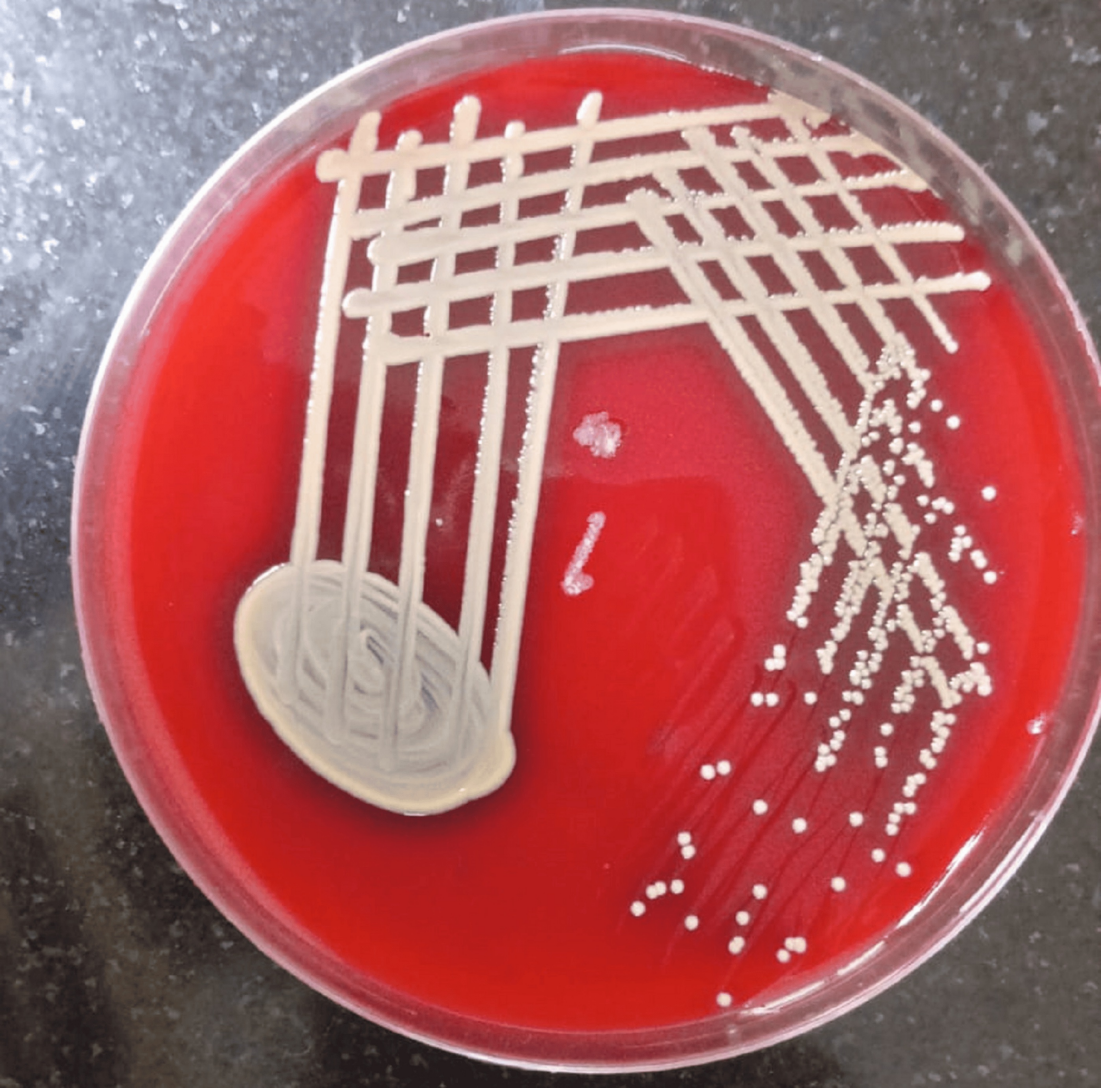
Staphylococcus aureus on blood agar

A total of 137 clinically diagnosed cases of active CSOM were included in the study. The maximum number of patients, i.e., 78 (56.9%), belonged to the 18-30-year age group followed by 29 (21.2%) patients in the 31-40-year age group, 20 (14.6%) patients in the 41-50-year age group, and 10 (7.3%) patients in the 51-60-year age group. The second and third decades of life had the maximum prevalence of COM. Female patients suffered slightly more than males as the female-to-male ratio was 1.28:1. Majority of patients, i.e., 129 (94%), had shown growth of microorganisms, while only eight (6%) patients had no growth in the culture. Out of 129 culture-positive cases, *Pseudomonas aeruginosa* (Figure 1) was the most commonly (33.6%, 46) isolated organism followed by *Staphylococcus aureus* (Figure 2) (16.8%, 23) in the study. Methicillin-resistant *Staphylococcus aureus* (MRSA) was isolated in 13.1% (18), and no growth was observed in 5.8% (8) of the subjects as shown in Table 1. 

**Table 1:** Organism-wise distribution of culture isolates (n=129)

S. no.	Organism	Number (n=129)	Percentage (%)
1	Pseudomonas aeruginosa	46	33.6
2	Staphylococcus aureus	23	16.8
3	Methicillin-resistant *Staphylococcus aureus*	18	13.1
4	Staphylococcus epidermidis	7	5.1
5	Klebsiella pneumoniae	6	4.4
6	Proteus mirabilis	6	4.4
7	Staphylococcus haemolyticus	4	2.9
8	Achromobacter denitrificans	2	1.5
9	Acinetobacter baumannii	2	1.5
10	Citrobacter koseri	2	1.5
11	Escherichia coli	2	1.5
12	Enterobacter asburiae	2	1.5
13	Staphylococcus capitis	2	1.5
14	Enterococcus faecalis	1	0.7
15	Klebsiella oxytoca	1	0.7
16	Morganella morganii	1	0.7
17	Pseudomonas putida	1	0.7
18	Serratia marcescens	1	0.7
19	Staphylococcus auricularis	1	0.7
20	Staphylococcus lugdunensis	1	0.7

The antibiotic susceptibility pattern of these organisms was studied, and *Pseudomonas aeruginosa* showed maximum sensitivity to piperacillin/tazobactam in 32 isolates (84.2%) followed by imipenem (72.7%). Maximum resistance was shown by *Pseudomonas aeruginosa* to levofloxacin (60.5%) followed by ciprofloxacin (56.5%) and ceftazidime (26.5%), as depicted in Table 2.

**Table 2:** Antimicrobial susceptibility pattern of Pseudomonas aeruginosa

S. no.	Antimicrobial susceptibility testing	Sensitive (%)	Intermediate (%)	Resistant (%)
1	Piperacillin/tazobactam	32 (84.2%)	4 (10.5%)	2 (5.3%)
2	Ceftazidime	31 (68.9%)	2 (4.4%)	12 (26.7%)
3	Cefoperazone/sulbactam	27 (61.4%)	10 (22.7%)	7 (15.9%)
4	Cefepime	31 (72.1%)	7 (16.3%)	5 (11.6%)
5	Imipenem	32 (72.7%)	1 (2.3%)	11 (25%)
6	Meropenem	31 (72.1%)	2 (4.7%)	10 (23.3%)
7	Ciprofloxacin	17 (37%)	3 (6.5%)	26 (56.5%)
8	Levofloxacin	15 (34.9%)	2 (4.7%)	26 (60.5%)

Out of a total of 23 cases of *Staphylococcus aureus*, 100% sensitivity was shown to linezolid and daptomycin followed by vancomycin and tetracycline in 90.9% of the isolated organisms. There was minimum sensitivity of isolates to ciprofloxacin (4.5%), as shown in Table 3.

**Table 3:** Antimicrobial susceptibility pattern of Staphylococcus aureus

S. no.	Antimicrobial susceptibility testing	Sensitive (%)	Intermediate (%)	Resistant (%)
1	Gentamicin	19 (86.4%)	0 (0%)	3 (13.6%)
2	Ciprofloxacin	1 (4.5%)	1 (4.5%)	20 (90.9%)
3	Levofloxacin	2 (9.1%)	0 (0%)	20 (90.9%)
4	Trimethoprim/sulfamethoxazole	11 (50%)	0 (0%)	11 (50%)
5	Erythromycin	8 (36.4%)	1 (4.5%)	13 (59.1%)
6	Clindamycin	17 (77.3%)	0 (0%)	5 (22.7%)
7	Linezolid	22 (100%)	0 (0%)	0 (0%)
8	Daptomycin	21 (100%)	0 (0%)	0 (0%)
9	Vancomycin	20 (90.9%)	1 (4.5%)	1 (4.5%)
10	Tetracycline	20 (90.9%)	0 (0%)	2 (9.1%)

MRSA showed 100% sensitivity towards minocycline, linezolid, and daptomycin followed by tetracycline (88.2%) and vancomycin (82.4%). None of the isolated organisms was sensitive to ciprofloxacin and levofloxacin, as depicted in Table 4. 

**Table 4:** Antimicrobial susceptibility pattern of MRSA MRSA: methicillin-resistant *Staphylococcus aureus*

S. no.	Antimicrobial susceptibility testing	Sensitive (%)	Intermediate (%)	Resistant (%)
1	Gentamicin	8 (47.1%)	3 (17.6%)	6 (35.3%)
2	Ciprofloxacin	0 (0%)	0 (0%)	17 (100%)
3	Levofloxacin	0 (0%)	0 (0%)	17 (100%)
4	Minocycline	1 (100%)	0 (0%)	0 (0%)
5	Trimethoprim/sulfamethoxazole	9 (52.9%)	0 (0%)	8 (47.1%)
6	Erythromycin	3 (17.6%)	0 (0%)	14 (82.4%)
7	Clindamycin	10 (58.8%)	0 (0%)	7 (41.2%)
8	Linezolid	15 (100%)	0 (0%)	0 (0%)
9	Daptomycin	13 (100%)	0 (0%)	0 (0%)
10	Vancomycin	14 (82.4%)	0 (0%)	3 (17.6%)
11	Tetracycline	15 (88.2%)	0 (0%)	2 (11.8%)

*Pseudomonas aeruginosa *(32.6%) was the most commonly isolated organism in the mucosal type of COM followed by *Staphylococcus aureus* (19.6%), MRSA (9.8%), *Staphylococcus epidermidis *(5.4%), *Klebsiella pneumoniae *(4.3%), and *Proteus mirabilis* (3.3%). Culture reports in cases of squamosal COM showed that *Staphylococcus aureus* was the most commonly isolated organism (including *Staphylococcus aureus* 12.8% with MRSA 23.1%) followed by *Pseudomonas aeruginosa* (30.8%), *Proteus mirabilis *(7.7%), *Staphylococcus epidermidis* (5.1%), and *Klebsiella pneumoniae *(5.1%). In patients with mucosal COM in one ear and squamosal COM in another ear and the sample being collected from mucosal COM, the most commonly isolated organism was *Pseudomonas aeruginosa* (66.7%) followed by *Staphylococcus epidermis* (16.7%) and *Staphylococcus capitis* (16.7%) as demonstrated in Table 5.

**Table 5:** Association between disease and isolated organisms MRSA: methicillin-resistant *Staphylococcus aureus*

S. no.	Isolated organism	Disease			P-value
		Mucosal (n=92)	Squamosal (n=39)	Mucosal/squamosal (n=6)	0.579
1	Pseudomonas aeruginosa	30 (32.6%)	12 (30.8%)	4 (66.7%)	
2	Staphylococcus aureus	18 (19.6%)	5 (12.8%)	0 (0%)	
3	MRSA	9 (9.8%)	9 (23.1%)	0 (0%)	
4	No growth	6 (6.5%)	2 (5.1%)	0 (0%)	
5	Staphylococcus epidermidis	7 (7.6%)	1 (2.6%)	1 (16.7%)	
6	Klebsiella pneumoniae	4 (4.3%)	2 (5.1%)	0 (0%)	
7	Proteus mirabilis	3 (3.3%)	3 (7.7%)	0 (0%)	
8	Staphylococcus haemolyticus	4 (4.3%)	0 (0%)	0 (0%)	
9	Achromobacter denitrificans	1 (1.1%)	1 (2.6%)	0 (0%)	
10	Acinetobacter baumannii	1 (1.1%)	1 (2.6%)	0 (0%)	
11	Citrobacter koseri	2 (2.2%)	0 (0%)	0 (0%)	
12	Escherichia coli	2 (2.2%)	0 (0%)	0 (0%)	
13	Enterobacter asburiae	2 (2.2%)	0 (0%)	0 (0%)	
14	Staphylococcus capitis	0 (0%)	1 (2.6%)	1 (16.7%)	
15	Enterococcus faecalis	0 (0%)	1 (2.6%)	0 (0%)	
16	Klebsiella oxytoca	0 (0%)	1 (2.6%)	0 (0%)	
17	Morganella morganii	1 (1.1%)	0 (0%)	0 (0%)	
18	Pseudomonas putida	1 (1.1%)	0 (0%)	0 (0%)	
19	Serratia marcescens	1 (1.1%)	0 (0%)	0 (0%)	
20	Staphylococcus auricularis	1 (1.1%)	0 (0%)	0 (0%)	
21	Staphylococcus lugdunensis	1 (1.1%)	0 (0%)	0 (0%)	

## Discussion

COM is one of the most commonly encountered diseases in ENT OPD. It is one of the main preventable causes of hearing loss [4]. Squamosal COM, in the long term, can lead to potentially life-threatening complications. Hence, knowing the antimicrobial pattern and drug susceptibility can help in guiding appropriate antibiotics and help in preventing the development of multidrug-resistant bacteria. The peak age group of patients suffering from active COM was 18-30 years (56.9%), which is similar to the study conducted by Bisht et al. [10] (48.66%) in the state of Uttarakhand in 2012. This result is also in concordance with a study conducted by Vishwanath et al. [11] in 2009 among randomly selected patients between one and 78 years of age in which the majority of patients (22.3%) were in the age group of 21-30 years. In a study by Mallick et al. [12] in 2016-2017, the majority of COM was observed in the 16-35-year age group. This could result from more frequent outdoor activities and exposure to environmental agents in the younger age groups. In the present study, females (56.2%) were more commonly affected than males (43.8%), with a female-to-male ratio of 1.28:1, which is similar to the study conducted by Bisht et al. [10] (1.20:1) and Prakash et al. [8] (1.2:1). However, in a study conducted by Garima et al. [13] in 2014-2015, the male-to-female ratio was 1.2:1, and in that by Kaur et al. [14], the male-to-female ratio was 1.53:1. No specific reason could be found out in the various studies in the past as well as in recent times. Out of 137 subjects, 129 (94%) of the subjects were culture positive and eight (6%) were culture negative. This observation is similar to the study conducted by Smitha et al. [15], with 91.15% culture positivity, and the study conducted by Mallick et al. [12], with 93.2% of the samples showing culture positivity. No bacterial growth in culture could result from other aetiologies like viral, fungal, or mixed infection. The most commonly isolated organism was *Pseudomonas aeruginosa *(33.6%), followed by *Staphylococcus aureus* (17.8%), MRSA (13.1%), *Staphylococcus epidermis* (5.1%), and *Klebsiella pneumoniae *(4.4%). However, no growth was seen in 6% of the subjects. Similar observations were made by Vishwanath et al. [11] in 2009, where *Pseudomonas aeruginosa* (32.2%) was the most commonly isolated organism, followed by *Staphylococcus aureus* (17.4%), coagulase-negative *Staphylococcus *spp. (8.7%), and *Klebsiella pneumoniae* (6.9%). This study is also in concordance with the one conducted by Wan Draman et al. [16] in 2018-2019 in Malaysia, among 81 patients suffering from COM where *Pseudomonas aeruginosa *(32.6%) was the most commonly isolated organism, followed by *Staphylococcus aureus* (16.9%). However, a study conducted by Bisht et al. [10] in the state of Uttarakhand demonstrated *Staphylococcus aureus* (52.63%) as the most commonly isolated organism, followed by *Pseudomonas aeruginosa *(24.56%). Another study conducted in Uttarakhand by Prakash et al. [8] demonstrated *Staphylococcus aureus *(48.69%) as the most commonly isolated organism, followed by *Pseudomonas aeruginosa* (19.89%). If we compare our study with studies done in other parts of the world, it is seen that isolated pathogens also vary depending upon the area of the study, whether rural or urban, immunocompromised or immunocompetent individuals, availability of medical facilities, as well as the antibiotic prescribing pattern of the attending physician. A cross-sectional study was conducted at Ayder Referral Hospital, Northern Ethiopia, among 162 cases of otitis media/acute otitis media by Wasihun and Zemene [17] in 2014-2015. A total of 216 bacteria were isolated from 157 (98.2%) patients. *Staphylococcus aureus* (46, 28.4%) was the most commonly isolated bacteria, followed by *Proteus mirabilis* (39, 24.1%), *Pseudomonas aeruginosa *(27, 16.7%), *Klebsiella *spp. (11.1%), and *Haemophilus influenzae* (18, 11.1%). A cohort study was conducted by Karn et al. [18] in Nepal in 2020 among 117 patients suffering from COM. *Pseudomonas aeruginosa* (80%) was the most common isolated organism, followed by *Staphylococcus aureus *(16%). 

In 2016, a cohort study was conducted in rural areas of South Africa by Toman et al. [19] to study the role of routine culture in treating CSOM. Among 14 patients suffering from COM, six were HIV positive, while the status of the other eight patients was not known. A total of 18 middle ear swabs were collected, and *Staphylococcus aureus *(5) was the most commonly isolated organism, followed by *Pseudomonas aeruginosa* (3), *Proteus mirabilis* (2), and *Providencia stuartii* (1). Five patients did not have any growth in the culture. The change in clinico-bacteriological profile could be due to the development of more resistant strains of the isolated organisms and changes in geography and climatic conditions over time. In our study, *Pseudomonas aeruginosa *was 84.2% sensitive to piperacillin/tazobactam. Sensitivity to imipenem was 72.7% (32), followed by 72.1% (31) sensitivity to meropenem and cefepime each. High sensitivity to cefoperazone/sulbactam was observed in 61.4% (27), while intermediate sensitivity was observed in 22.7% (10) of the isolated organisms. High sensitivity to amikacin was observed in 57.8% (26) and intermediate sensitivity in 2.2% (1). About 34.9% of isolates had high sensitivity and 4.7% had intermediate sensitivity to levofloxacin. The maximum resistance of *Pseudomonas aeruginosa* was seen to antibiotics like levofloxacin (60.5%) and ciprofloxacin (56.5%). A similar observation was made by Garima et al. [13] in 2014-2015, where high sensitivity of *Pseudomonas aeruginosa* was observed to piperacillin/tazobactam (83.8%), cefoperazone/sulbactam (83.8%), meropenem (73.9%), imipenem (71%), and cefepime (71.7%). However, in contrast to our study, high sensitivity of the organism was also observed to amikacin (74.7%) and levofloxacin (63.6%). Decreasing sensitivity to levofloxacin could be a result of easy accessibility to over-the-counter medications for COM and injudicious use of the drug, which could lead to the development of antibiotic resistance in the bacteria. *Staphylococcus aureus* showed 100% sensitivity to linezolid and daptomycin, followed by high sensitivity (90.5%) and intermediate sensitivity (4.8%) to vancomycin and 90.5% sensitivity to tetracycline. Sensitivity to gentamicin was seen in 85.7% of the isolated organisms, with maximum resistance to ciprofloxacin (90.5%) and levofloxacin (90.5%). This is in concordance with a study conducted by Goyal et al. [20] in 2021-2022 with 100% sensitivity to linezolid and vancomycin, 94.4% sensitivity to tetracycline, and 86.4% sensitivity to gentamicin. However, in contrast to our study, 45.5% of the isolated organisms were sensitive to ciprofloxacin. MRSA showed 100% sensitivity towards minocycline, linezolid, and daptomycin, each followed by tetracycline (88.2%) and vancomycin (82.4%). None of the isolated organisms were sensitive to ciprofloxacin or levofloxacin. Resistance to ciprofloxacin in *Staphylococcus* species could be due to inappropriate use of the drug as the first-line agent in chronically discharging ears instead of culture-guided antibiotics. Immunoglobulins (Ig) such as IgG, IgA, and secretory IgA play an important role in guarding against CSOM. IgA helps prevent colonization and bacterial attachment to the middle ear mucosa. IgG activates the complement pathway and helps in the phagocytosis of pathogenic bacteria [21]. More studies need to be done to understand COM's pathogenicity and the effects of host immune factors on disease progression. More effective treatment strategies need to be developed for multidrug-resistant bacteria. A study conducted by Muhammad et al. [22] in Iraq has studied the use of bacteriophage in treating experimentally infected rabbits' ears. The infected lesion healed rapidly when treated with bacteriophage, unlike topical antibiotics. 

The distribution of isolated organisms between mucosal and squamosal COM resulted in a p-value of 0.579, which is not statistically significant. This is similar to the study conducted by Kombade et al. [23], in which organisms were distributed between mucosal and squamosal COM types and resulted in a p-value of 0.542, which was not statistically significant. *Pseudomonas aeruginosa* (32.6%) was the most commonly isolated organism in mucosal COM, followed by *Staphylococcus aureus *(19.6%), MRSA (9.8%), *Staphylococcus epidermidis* (5.4%), *Klebsiella pneumoniae* (4.3%), and *Proteus mirabilis* (3.3%). In a study conducted in 2018 by Singh et al. [24] in patients of mucosal COM, *Staphylococcus aureus* (43.2%) was the most commonly isolated organism followed by *Pseudomonas aeruginosa *(27.9%), coagulase-negative *Staphylococcus aureus* (9.3%), *Klebsiella pneumoniae* (6.8%), and *Proteus *species(5.08%). This changing pattern of causative organisms may result from changes in environmental conditions and geography. Squamosal COM showed *Staphylococcus aureus* (12.8%) with MRSA (23.1%) as the most commonly isolated organism, followed by *Pseudomonas aeruginosa* (30.8%), *Proteus mirabilis *(7.7%), *Staphylococcus epidermidis* (5.1%), and *Klebsiella pneumoniae *(5.1%). These results are in concordance with a study conducted by Shrivastava et al. [25], which showed *Staphylococcus aureus *as the most commonly isolated organism, followed by *Pseudomonas aeruginosa*, *Klebsiella*, and *Proteus mirabilis.*

In patients with mucosal COM in one ear and squamosal COM in another ear, with the sample being collected from mucosal COM, the most commonly isolated organism was *Pseudomonas aeruginosa *(66.7%), followed by *Staphylococcus epidermis* (16.7%) and *Staphylococcus capitis* (16.7%). However, no significant study has been done in patients with mucosal COM in one ear and squamosal disease in another disease. More studies are required to study the change in antibiotic susceptibility patterns in such patients and the effects of mucosal COM on squamosal COM and vice versa. This is due to the fact that the nasopharynx may serve as a common site for the mixing of pathogenic bacteria from both ears via the eustachian tube.

Limitations

Only aerobic culture of isolated organisms was done. Hence, data regarding anaerobes could not be collected. To confirm our findings and assess the effectiveness of culture-guided antibiotics, multicentric studies with larger sample sizes and longer follow-up periods are needed.

## Conclusions

COM is one of the most commonly encountered diseases in ENT OPD. The most commonly affected age group was observed in the second to third decades of life with no significant male-to-female predisposition. *Pseudomonas aeruginosa *was the most commonly isolated organism followed by *Staphylococcus aureus.* *Pseudomonas aeruginosa* demonstrated susceptibility to piperacillin/tazobactam and meropenem, while *Staphylococcus aureus* was susceptible to linezolid, daptomycin, and vancomycin. Both demonstrated maximum resistance to ciprofloxacin and levofloxacin. The easy availability of over-the-counter ear drops along with poor follow-up of patients resulting in indiscriminate and incomplete course of treatment leads to the development of multidrug-resistant bacteria. Hence, there is an urgent need to study the bacteriological profile and antimicrobial susceptibility pattern in COM on a regional basis and design the antibiotic policy of every region based on their antibiogram. This would help the clinician guide appropriate antibiotic treatment at distant places where laboratory facilities are unavailable.

## References

[1] Wackym PA, Snow Jr JB, Chole RA, Nason R (2016). Chronic otitis media. Ballenger's Otorhinolaryngology: Head and Neck Surgery.

[2] Flint PW, Haughey BH, Lund V (2010). Chronic otitis media, mastoiditis, and petrositis. Cummings Otolaryngology: Head and Neck Surgery.

[3] Benson J, Mwanri L (2012). Chronic suppurative otitis media and cholesteatoma in Australia's refugee population. Aust Fam Physician.

[4] Acuin J (2004). Chronic suppurative otitis media: burden of illness and management options. Chronic suppurative otitis media: burden of illness and management options.

[5] Mittal R, Lisi CV, Gerring R (2015). Current concepts in the pathogenesis and treatment of chronic suppurative otitis media. J Med Microbiol.

[6] Yadav K, Kaushik S, Rani K, Tyagi AK (2021). Bacterial profile and antimicrobial susceptibility pattern of chronic suppurative otitis media from a tertiary care hospital in Kannauj, Uttar Pradesh, India. J Clin Diagn Res.

[7] Bhumbla U, Gupta P, Mathur DR (2016). Current trends in microbial profile and resistance pattern in CSOM in a semiurban hospital of Southern India. J Evol Med Dent Sci.

[8] Prakash R, Juyal D, Negi V, Pal S, Adekhandi S, Sharma M, Sharma N (2013). Microbiology of chronic suppurative otitis media in a tertiary care setup of Uttarakhand state, India. N Am J Med Sci.

[9] Holland TL, Woods CW, Joyce M (2009). Antibacterial susceptibility testing in the clinical laboratory. Infect Dis Clin North Am.

[10] Bisht RS, Sikarwar V, Pal S, Jakhwal C (2014). Microbiological evaluation of active chronic otitis media at Base Hospital Srikot, Uttarakhand. J Evol Med Dent Sci.

[11] Vishwanath S, Mukhopadhyay C, Prakash R, Pillai S, Pujary K, Pujary P (2012). Chronic suppurative otitis media: optimizing initial antibiotic therapy in a tertiary care setup. Indian J Otolaryngol Head Neck Surg.

[12] Mallick A, Sharma H, Mishra AK, Maggon NV, Sethi A (2018). Bacteriological profile and antibiotic resistance in cases of chronic otitis media and its clinical implications. Int J Otorhinolaryngol Head Neck Surg.

[13] Garima Garima, Chaurasia D, Poorey VK (2016). Antimicrobial susceptibility pattern of bacterial isolates from chronic suppurative otitis media patients in Central India. Indian J Microbiol Res.

[14] Kaur P, Sood AS, Sharma S, Awal G (2018). Microbiological profile and antimicrobial susceptibility pattern of chronic suppurative otitis media in a tertiary care centre. Trop J Pathol Microbiol.

[15] Smitha NR, Jnaneshwara KB, Patil AB, Harshika YK, Medegar S (2018). A study of aerobic bacteriological profile of chronic suppurative otitis media in a tertiary care hospital, South India. Indian J Microbiol Res.

[16] Wan Draman WN, Md Daud MK, Mohamad H, Hassan SA, Abd Rahman N (2021). Evaluation of the current bacteriological profile and antibiotic sensitivity pattern in chronic suppurative otitis media. Laryngoscope Investig Otolaryngol.

[17] Wasihun AG, Zemene Y (2015). Bacterial profile and antimicrobial susceptibility patterns of otitis media in Ayder Teaching and Referral Hospital, Mekelle University, Northern Ethiopia. Springerplus.

[18] Karn RR, Acharya R, Rajbanshi AK (2021). Antibiotic resistance in patients with chronic ear discharge awaiting surgery in Nepal. Public Health Action.

[19] Toman J, Moll A, Barnes M, Shenoi S, Porterfield JZ (2019). The role of routine culture in the treatment of chronic suppurative otitis media: implications for the standard of care in rural areas of South Africa. Trop Med Infect Dis.

[20] Goyal D, Pal N, Agrawal Y, Hooja S, Sharma R (2024). Bacteriological profile and antibiotic susceptibility pattern of CSOM at a tertiary care hospital. RUHS J Health Sci.

[21] Khairkar M, Deshmukh P, Maity H, Deotale V (2023). Chronic suppurative otitis media: a comprehensive review of epidemiology, pathogenesis, microbiology, and complications. Cureus.

[22] Muhammad MI, Sawa MI, Dizaye KF (2011). Lytic activity of bacteriophage against Pseudomonas aeruginosa isolated fro Patients with chronic suppurative otitis media. Lytic activity of bacteriophage against Pseudomonas aeruginosa isolated from patients with chronic suppurative otitis media (CSOM) in Erbil/Iraq.

[23] Kombade SP, Kaur N, Patro SK, Nag VL (2021). Clinico-bacteriological and antibiotic drug resistance profile of chronic suppurative otitis media at a tertiary care hospital in Western Rajasthan. J Family Med Prim Care.

[24] Singh BR, Pradhan S, Murthy R, Agrawal E, Barapatre R, Kumari N, Pandey A (2019). Emergence of antibiotic resistance in bacteria isolated from tubotympanic type of chronic suppurative otitis media in Chhattisgarh. Int J Otorhinolaryngol Head Neck Surg.

[25] Shrivastava U (2023). Sensitivity and resistance patterns of antimicrobials in chronic otitis media squamosal. Int J Otorhinolaryngol Clin.

